# Reviewed article: Lerm BR, Pletsch V, Géa MFF, Santos IS, Regner AP, Giugliani C. Social vulnerability of children and adolescents who are victims of sexual violence assisted in a specialized service: descriptive study, Porto Alegre and metropolitan region, 2018. Epidemiol Serv Saude. 2026:35;e20240780

**DOI:** 10.1590/S2237-96222026v35e20240780.a

**Published:** 2026-10-30

**Authors:** Marimeire Morais da Conceição

**Affiliations:** 1 Secretaria da Saúde do Estado da Bahia, Salvador, BA, Brazil Secretaria da Saúde do Estado da Bahia Salvador BA Brazil

## Peer review A

## Questionnaire

Does the manuscript contain new and significant information to justify publication? Yes

Does the Abstract (Summary) clearly and accurately describe the content of the article? No

Is the problem significant and concisely stated? Yes

Are the methods described comprehensively? Yes

Are the interpretations and conclusions justified by the results? No

Is adequate reference made to other work in the field? Yes

## Manuscript Structure:

Length of article is: Adequate

Number of tables is: Adequate

Number of figures is: Adequate

Please state any conflict(s) of interest that you have in relation to the review of this paper: None

Interest: Excellent

Quality: Average

Originality: Good

Overall: Good

Recommendation: Major Revision

## Comments

Prezados editores e autores,

Inicio este parecer cumprimentando a todos com estima e declaro minha satisfação em poder colaborar com a qualificação deste manuscrito. Também, diante do esforço demonstrado pelos autores, devo parabenizá-los pelo estudo realizado.

Embora seja visível a importância temática e dedicação pela construção do material apresentado, acrescento sugestões para que algumas alterações sejam realizadas, na intenção de contribuir com a melhoria da qualidade do material.

Att.,

Avaliador(a)

+++++++++++++++++++++++++++++++++++++++++++++++++++++++++

ANÁLISE GERAL: Em geral, é necessária uma ampla e minuciosa revisão textual (gramática, pontuação, ortografia). apesar do adequado encadeamento das ideias, da construção lógica e sequenciada. Abaixo, listo sugestões para o aprimoramento do trabalho, no intuito de possibilitar uma futura publicação.

Alguns trechos do texto que precisam ser revistos foram sinalizados em vermelho.

TÍTULO

Inadequado: título extenso, sugiro reduzir para até 16 palavras, é preciso adequar segundo o objetivo, sugiro:

Violência sexual contra crianças e adolescentes: análise usando georreferenciamento de casos no Rio Grande do Sul

Ou

Violência sexual infantojuvenil: georreferenciamento de casos atendidos no Rio Grande do Sul, Brasil

Obs.: o título apresenta o ano de 2022, porém no resumo os autores apontam 2018. É preciso definir qual o ano de registro dos dados. Caso as normas da resvista exijam, acrescentar o ano no título

RESUMO: precisa ser revisto quanto às normas da Revista

Objetivo: sugiro adequar para: Descrever o perfil e o contexto social de crianças/adolescentes vítimas de violência sexual atendidos em um serviço de referência do Rio Grande do Sul

Obs.: Atentar para o ano real: é 2018 ou 2022? O objetivo está diferente do objetivo da introdução

Método: acrescentar o ano real dos dados (2018 ou 2022?)

Resultados: incluir percentual em “e vítimas de violência intrafamiliar.”. Revisar ortografia e pontuação

Conclusão: precisa ser mais pontual e específico, pois qualquer estudo epidemiológico serva no subsídio a politicas públicas

Descritores: “Violência Sexual”; “Abuso Sexual de Crianças e Adolescentes” não são descritores controlados no DeCS, sugiro adequação conforme: https://decs.bvsalud.org/

INTRODUÇÃO:

Precisa de melhor encadeamento lógico

O conceito de violência sexual entre aspas está escrito ipsi literis, por isto precisa ter a citação adequada segundo as normas da revista

O termo “infanto-juvenil” perdeu o hifem a partir do novo acordo ortográfico, assim deve ser substituído no texto por “infantojuvenil”, ou seja, sem hifem

Parágrafos com mais de 08 linhas devem ser evitados, por favor, adequar

A frase “não há registro de estudos que o tenham utilizado para investigar o contexto social de crianças e adolescentes vítimas de violência sexual” é comprometedora, pois os autores não conhecem a totalidade dos estudos publicados.... sugiro alterar o texto para “há escassez de estudos que o tenham utilizado para investigar o contexto social de crianças e adolescentes vítimas de violência sexual”.

A introdução precisa de mais argumentação e apresentar lacunas da ciência que justifiquem a realização do estudo

O objetivo “caracterizar o perfil das crianças e adolescentes atendidos em um serviço de referência para atenção às vítimas de violência sexual no estado do Rio Grande do Sul.” Está diferente do objetivo do resumo e do

Sugiro que no último parágrafo escrevam a relevância do estudo

Obs: Evitar parágrafos com uma única frase, com períodos com mais de 3 linhas e parágrafos extensos (acima de 8 linhas)

MÉTODOS

Precisa ser reorganizado, sugiro seguir:

• Delineamento

Parcialmente adequado, deve limitar-se a dizer o tipo de estudo, as demais informações devem ir para o contexto

OBS: o texto não menciona o uso de nenhum guia de relatório de pesquisa ou check list para reportar resultados de pesquisa como o STROBE. Sugere-se usar o STROBE

• Contexto

Deve-se retirar o nome do serviço, pois a resolução 580/2018 do Conselho Nacional de Saúde exige que o pesquisador resguarde a identidade dos serviços públicos do SUS

Sugiro excluir as características do serviço, pois ele não é objeto de estudo

• Participantes

Sugiro alterar, pois os dados são extraídos de prontuários e não diretamente das crianças e adolescentes. Sugiro “Foram extraídos dados de prontuários com os seguintes critérios de inclusão xxxxxx e exclusão: xxxxxx”

Houve calculo amostral?

• Coleta de dados

Nada digno de nota

• Período do estudo

Sugiro que o período de coleta de dados esteja no perimeiro parágrafro da coleta de dados

• Questão de pesquisa ou hipóteses

Não foram apresentas

• Tratamento e Seleção das variáveis

Adequados

• Análse dos dados

Não cita referencial de análise dos dados, adequadar

• Aspectos éticos

Sugiro retirar o nome do hospital devido a questões éticas da pesquisa em serviços do SUS

++++++++++++++++++++++++

RESULTADOS

Na Tabela 2 sugiro remover a coluna da fonte de dados, visto que após remoção de duplicados, perde-se a fonte de onde o texto que permaneceu é oriundo.

Padronizar o título das tabelas

Na Tabela 2, questiono: os estudos são publicados somente em Português? Estes estudos nacionais não têm a versão para outros idiomas?

DISCUSSÃO

Precisa apresentar inicialmente os resultados extraídos do estudo que serão discutidos na ordem em que esta discussão será feita, sem apresentadas dados numéricos. Sugiro que falem da questão da vulnerabilidade pois é o maior diferencial deste estudo

A frase “fator que pode ter retardado a busca por ajuda,” é perigosa pois não tem a sustentação nem dos dados apresentados nem de referências citadas pelos autores

A discussão vai e volta na mesma questão: o sexo das vítimas, sugiro que, a partir de “Diversos estudos” sejam excluídas as considerações e substituídas pela questão da vulnerabilidade

O texto “A violência sexual infanto-juvenil, com exceção do tipo exploração sexual - que é, sem dúvida, mais frequente entre a população mais vulnerável (26)” tem uma assertividade que pode comprometer os autores, é necessário ponderar a forma de escrever o texto para evitar questionamentos futuros.

A frase “devido à escassez de estudos de base populacional” é contradita no parágrafo subsequente

Existem repetições desnecessárias de dados como “Observou-seque a maior parte das meninas vivia em áreas de maior vulnerabilidade do que os meninos.”, sugiro rever

Sugiro não usar o termo “combate” para representar medidas que visam coibir a violência sexual infantojuvenil, o termo “enfrentamento” é mais adequado pois trata de um conjunto de medidas preventivas, interventivas e de cuidados às vítimas e toda a sociedade

A discussão está superficial e não apresenta argumentações para além do que já está publicado. É necessário inovar na discussão, principalmente por ter apresentado resultados inovadores

Sugiro confrontar os resultados encontrados com a literatura internacional mais recente. A temática é muito explorada, no entanto os autores utilizaram referências antigas. De preferência, os textos citados precisam ter até 05 anos de publicação, exceto para materiais únicos como o relatório mundial da violência publicado pela OMS em 2015 e a linha do cuidado do Ministério da saúde publicado em 2011

World Health Organization (WHO). Global status report on violence prevention 2014[Internet]. 2015 [cited 2018 Jul 06]. Available from: https://apps.who.int/iris/bitstream/handle/10665/145087/WHO_NMH_NVI_14.2_eng.pdf?sequence=3

Limitações do estudo

Nada a considerar

REFERÊNCIAS

Padronizar a apresentação das referências de acordo com a RESS

Substituir referências antigas por referências recentes (sugiro usar no mínimo 70% de referências de estudos publicados de 2020 à 2025 pois demostra discussões mais atuais)

Apresentar ao menos uma referência na metodologia além da referência do STROBE vide: https://www.strobe-statement.org/checklists/

+++++++++++++++++++++++

Sugiro seguir o check list STROBE para verificação de elementos necessários para reportar com qualidade estudos transversais.

É o meu parecer

Att.,

Avaliador(a) 

